# Clinical Profile of Dengue Infection in Patients with Hematological Diseases

**DOI:** 10.4084/MJHID.2011.039

**Published:** 2011-09-08

**Authors:** Sanjeev Kumar Sharma, Tulika Seth, Pravas Mishra, Nitin Gupta, Narendra Agrawal, Shobha Broor, M Mahapatra, Renu Saxena

**Affiliations:** 1 Department of Hematology and; 2 Microbiology, All India Institute of Medical Sciences, New Delhi, India

## Abstract

Managing hematological disorders in a tropical country presents several unique diagnostic and management problems. Apart from the disease process, we need to be aware of infections that can exacerbate or mimic serious hematological problems. We present here a series of five patients with pre-existing hematological diseases who were infected by dengue virus. These cases highlight the need to keep a strong suspicion of common endemic diseases in tropical countries before considering extensive workup for the basic hematological disease. There was no mortality and all patients recovered without any significant impact on their pre-existing hematological condition inspite of their low baseline blood counts. There was no excessive bleeding, prolonged stay in the hospital or relapse of underlying hematological disease in these patients and the only major concern was the increased anxiety among both the patient and treating physician regarding the relapse/progression of pre-existing hematological disease.

## Introduction:

Patients with pre-existing hematological diseases are equally at risk of acquiring vector-borne infections as is the general population. The impact of dengue infection on the hematological patients has been rarely studied. Dengue infection causes leucopenia and thrombocytopenia and may mimic relapse or progression of an underlying hematological disease. Dengue virus infection has never been looked into seriously in hematological patients. We had a dengue epidemic in the months of September to November 2010 in New Delhi, India. This epidemic of dengue infection gave us the opportunity to study the impact of dengue in patients with pre-existing hematological diseases. All these patients presented to us at a time when dengue infection was rampant in the community.

## Case series

### Case 1:

A 4-year-old child, who had undergone a sibling matched allogeneic bone marrow transplant (BMT) for chronic myeloid leukemia-blast crises (CML-BC), presented to the emergency on day 180 of her transplant with high grade fever and bodyaches for 3 days and bleeding from nose for 1 day. Hemogram revealed low counts [hemoglobin (Hb) 10.6g/dl, total leukocyte count (TLC) 2.2x10^9^/l with differential count: neutrophils 42%, lymphocytes 48%, monocytes 8%, eosinophils 2% with no atypical cells, and platelet count 10x10^9^/l]. Relapse, graft rejection and sepsis were all considered as the cause of decreasing leukocyte and platelet counts in this post BMT patient. Chimerism was 100% a month back. There were no blasts in the peripheral smear and the bone marrow examination was normal. Blood cultures were sterile and a computerised tomography scan of chest and abdomen did not reveal any focus of infection. Broad spectrum antibiotics were started as per institutional policy for such patients and her general condition improved even though counts remained low. A quantitative polymerase chain reaction (PCR) for cytomegalovirus (CMV) was negative. The mu-capture enzyme linked immunosorbent assay (ELISA) for dengue IgM was positive and the child was managed conservatively with platelet transfusions. The counts recovered by day 9 after first onset of symptoms.

### Case 2:

A 10-year-old child, a case of immune thrombocytopenic purpura (ITP) on low dose steroids was maintaining his platelet counts at around 50 x10^9^/l, when he developed fever and petechial rash of 5 days duration with fall in platelets to 20x10^9^/l. With the previous case in mind and before considering change in treatment for ITP, dengue NS-1 antigen and later mu-capture ELISA for dengue IgM were performed and found to be positive. He was treated conservatively with intravenous fluids and antipyretics. Platelet counts started increasing on 7^th^ day of illness. The steroids were not stopped during this episode of fever. In patients of ITP presenting with fever and falling platelet counts for any reason, we even consider increasing the dose of steroids along with therapy for fever in case of bleeding or platelet counts below 20x10^9^/l. In this patient we continued with the same dose as platelets did not fall below 20x10^9^/l and the child did not have any evidence of serious bleed except for petechiae.

### Case 3:

A 35-year-old lady on maintenance therapy for acute promyelocytic leukemia (APL), developed high grade fever and petechial rash over limbs and bleeding from gums for 3 days. She had features of generalised bodyaches and malaise for a week prior to fever. Hemogram revealed Hb 11.2g%, TLC 4.8 x 10^9^/l with normal differential count and no atypical cells, and platelet count 8 x10^9^/l. Prothrombin time (test 12secs and control 13secs) and activated partial thromboplastin time (test 32secs and control 30secs) were normal. Pateint received antibiotics and single donor platelet transfusions to maintain platelet counts above 20 x10^9^/l. The mu-capture ELISA for dengue IgM was positive. Her platelet counts increased over the next 10 days to more than 150 x 10^9^/l. Bone marrow examination was not done as patient showed symptomatic improvement and there was no evidence of APL relapse on peripheral smear. However, a subsequent marrow aspirate done to document molecular response as per protocol confirmed absence of disease.

### Case 4:

A 50-year-old gentleman, a case of autoimmune hemolytic anemia (AIHA) diagnosed in 2007, was off steroids for the last two years and maintained hemoglobin levels between 8 to 10g/dl. He developed high grade fever with bodyaches for 1 week. Hemogram revealed Hb 7.4g/dl, TLC 3.3x10^9^/l and platelet count 40x10^9^/l. Workup for fever revealed positive dengue serology (IgM) by ELISA. There were no bleeding manifestations. Platelet counts increased to more than 150x 10^9^/l by 6^th^ day.

### Case 5:

An 8-years-old child, a case of thalassemia major, who had undergone a successful sibling matched allogeneic BMT presented on day 70 of transplant with high grade fever of 1 day duration. Hemogram showed Hb 8.1g/dl, TLC 1.4 x10^9^/l and platelet count 26 x10^9^/l. Mu-capture ELISA for dengue IgM on day 8 of admission was positive. The child’s fever subsided after 2 days but the counts continued to drop till day 5 of admission after which they started to rise.

## Results:

All of these patients presented with high grade fever and thrombocytopenia, though only three patients had bleeding manifestations. Leucopenia was also present in three of these patients. Other complaints were bodyaches and retro-orbital pain (Table 1).

All these patients had dengue IgM positive by ELISA implying that inspite of being affected by pre-existing hematological diseases/immunodeficient conditions they were able to mount antibody response to dengue virus (Table 2), though dengue IgG was not evaluated prospectively. Serotyping revealed dengue virus type 1 by PCR and culture in all five cases. Four of our five patients with dengue fever received inpatient treatment with intravenous fluids and/or platelet transfusion and one patient (with AIHA) was managed on outpatient basis.

None of the patients developed dengue shock syndrome or significant bleeding. There was no mortality in spite of severe thrombocytopenias in some of these cases implying that pre-existing hematological diseases do not put such patients at higher risk of morbidity or mortality if they contract dengue.

## Discussion:

Dengue is one of the most common mosquito-borne infectious diseases prevalent in tropical countries. It has been a major public health problem causing significant morbidity and mortality in the general population. In patients with pre-existing hematological diseases, dengue infection can pose a major diagnostic challenge because of similar clinical presentations with fever and cytopenias. Patients with hematological diseases like idiopathic thrombocytopenic purpura, leukemias and transplant recipients are equally vulnerable to dengue. There is no data on the impact of dengue in immunocompromised patients and those with hematological diseases, baring a few case reports.^1^,^2^

Dengue fever is caused by a positive single stranded RNA virus of the flaviviridae family. It is spread by Aedes mosquitoes (*Aedes aegyti and Aedes albopictus*). Leukopenia, thrombocytopenia, and hemorrhagic diathesis are the charasteristic hematologic findings in dengue virus infection. Leukopenia appears early in the course of illness and is thought to occur as a direct effect of dengue virus on the bone marrow.^3^ Dengue also causes a transient decrease in maturation of erythroid precursors,^4^ however because of the long half life of the red cells, dengue does not cause severe anemia in infected individuals. Cause of thrombocytopenia is multifactorial. Both bone marrow suppression and platelet destruction contribute to thrombocytopenia, with later playing more important role.^5^ Generation of virus-antibody immune complexes, leading to complement activation, is considered to be responsible for the platelet destruction.^6^,^7^ In patients with pre-existing hematological diseases the bone marrow reserve in already compromised and any further insult to hematopoeitic cells may have serious consequences. However, when we observed our patients prospectively, we did not find any increased morbidity or mortality, probably because of transient and temprorary effect of dengue virus on bone marrow, reflected by nearly complete recovery in all patients.

Clinically, dengue presents with fever, bodyaches, joint pains and bleeding manifestations in form of epistaxis, petechiae and gastrointestinal bleeding. All of the five patients had fever at presentation, though only three had bleeding manifestations. Laboratory diagnosis of dengue depends on the clinical stage of the patient. In the early stage of disease, fever and viremia are accompanied by NS1 (non structural protein 1) antigens in the blood. After a week dengue specific IgM and IgG antibodies appear in the blood.^8^ The capture ELISA for IgM detection, which was performed in all patients, is the most useful serologic procedure currently available and it is widely recommended for serological surveillance.^9^ There is no specific antiviral treatment or vaccine for dengue and the patients recovered after fluids and supportive therapy.

Though our series had small number of cases, the detailed study of dengue infection in patients with pre-existing hematological diseases gave us a new insight into the clinico-pathological impact of dengue in the clinical course of these diseases and the need to keep a high index of suspicion in diagnosis of such infections. Moreover, immunocompromised state did not seem to alter the clinical impact of dengue infection in patients with pre-existing hematological diseases. The serotype detected was dengue virus type 1, which causes a milder form of infection.

## Figures and Tables

**Table 1:** Characteristics of dengue infected patients with pre-existing hematological diseases.

Case N_o_.	Age (yrs)	Hematological disease	Clinical presentation	Diagnostic test	Lowest platelet count	Lowest TLC count	Platelet transfusion	Time to clinical recovery
1	4	CML-BC post BMT	Fever, epistaxis	IgM positive	10x10^9^/l	2.2x10^9^/l	required	9 days
2	10	ITP	Fever, petechiae	NS-1 & IgM positive	20x10^9^/l	4.6x10^9^/l	not required	7 days
3	35	APL	Fever, gum bleed	IgM positive	8x10^9^/l	4.8x10^9^/l	required	10 days
4	50	AIHA	Fever, bodyaches	IgM positive	40x10^9^/l	3.3x10^9^/l	not required	6 days
5	8	Thal major post BMT	Fever, retro-orbital pain	IgM positive	26x10^9^/l	1.4x10^9^/l	not required	12 days

CML-BC- chronic myeloid leukemia-blast crises, BMT-bone marrow transplant, ITP-idiopathic thrombocytopenic purpura, APL- acute promyelocytic leukemia, AIHA-autoimmune hemolytic anemia, Thal- thalassemia.

**Table 2:** Salient features observed in hematological patients infected with dengue virus.

- Clinical features were similar to those usually seen in patients without pre-existing hematological diseases - Dengue serology was positive in all cases and was not affected by underlying immunocompromised state. - A strong clinical suspicion averted the need for unnecessary and costlier investigations. - There was complete recovery of counts to baseline with supportive treatment.

## References

[1] Visuthranukul J, Bunworasate U, Lawasut P (2009). Dengue hemorrhagic fever in a peripheral blood stem cell transplant recipient: the first case report. Infect Dis Reports.

[2] Ullah K, Ahmed P, Raza S (2007). Allogeneic stem cell transplantation in hematological disorders: single center experience from Pakistan. Transplant Proc.

[3] Kalayanarooj S, Vaughn DW, Nimmannitya S (1997). Early clinical and laboratory indicators of acute dengue illness. J Infect Dis.

[4] Na-Nakorn S, Suingdumrong A, Pootrakul S, Bhamarapravati (1966). Bone-marrow studies in Thai haemorrhagic fever. Bull World Health Organ.

[5] Halstead SB (2007). Dengue. Lancet.

[6] Rigau-Pérez JG, Vorndam AV, Clark GG (2001). The dengue and dengue hemorrhagic fever epidemic in Puerto Rico, 1994–1995. Am J Trop Med Hyg.

[7] Mitrakul C, Poshyachinda M, Futrakul P, Sangkawibha N, Ahandrik S (1977). Hemostatic and platelet kinetic studies in dengue hemorrhagic fever. Am J Trop Med Hyg.

[8] WHO (1997). Dengue haemorrhagic fever: diagnosis, treatment, prevention and control.

[9] Nawa M, Takasaki T, Yamada KI, Akatsuka T, Kurane I (2001). Development of dengue IgM-capture enzyme-linked immunosorbent assay with higher sensitivity using monoclonal detection antibody. J Virol Methods.

